# Investigating the Differences in the Simple Reaction Time and Muscle Stiffness Between Gym Users and Open-Skills Sport Practitioners: An Exploratory Study

**DOI:** 10.3390/brainsci16060563

**Published:** 2026-05-26

**Authors:** Luca Petrigna, Alessandra Amato, Claudio Di Brigida, Salvatore Spinella, Giuseppe Evola, Giuseppe Musumeci

**Affiliations:** 1 Section of Anatomy, Histology and Movement Science, Department of Biomedical and Biotechnological Sciences, School of Medicine, University of Catania, Via S. Sofia 97, 95123 Catania, Italy; alessandra.amato@unict.it (A.A.); g.musumeci@unict.it (G.M.); 2 PhD Program in Sports Science, Catholic University of Murcia (UCAM), Universidad de Murcia, 30003 Murcia, Spain; 3 Centri Sportivi Aziendali e Industriali (CSAIn), 00144 Rome, Italy; 4 Department of Surgery, Garibaldi Hospital, 95121 Catania, Italy; 5 Research Center on Motor Activities (CRAM), University of Catania, Via S. Sofia 97, 95123 Catania, Italy

**Keywords:** processing speed, sEMG, cognitive function, risk of fall, physical activity

## Abstract

**Background/Objectives**: The number of people who practice gym activities is increasing. Most gym activities take place within a building, and the movements are controlled, making them closed-skill activities. This could decrease the processing speed capacity. The objective was to investigate whether a difference, assessed by a simple reaction time and muscle stiffness task, exists between people who practice gym versus open-skills sports activities. **Methods**: A total of 58 gym users and open-skills sport practitioners were recruited. Participants’ anthropometric characteristics were evaluated. Electrodes were set at the tibialis anterior (TA) and gastrocnemius lateralis (GL), and participants performed the simple reaction time task. A drop jump test (muscular stiffness) was also executed. A multiple comparison test was adopted to study the differences between groups for FAT%, reaction time, and ground contact time. The significance level was set at *p* ≤ 0.05. **Results**: Data from the groups presented no statistically significant differences in the simple reaction time task (*p* = 0.999) and in the drop jump (*p* = 0.999), or from a superficial electromyography point of view. **Conclusions**: This exploratory study detected no statistically significant differences between the groups. The study design does not support equivalence conclusions. Further studies are required to understand the topic in depth.

## 1. Introduction

Since 2014, the scientific literature has adopted the term fitness revolution [1]. In recent years, the number of people who attend gyms and fitness facilities has been increasing. While sport clubs practitioners are usually more interested in fun, social aspects, physical performance and competition, fitness center users are more focalized on health and weight control [2]. Activities in a gym context could require the use of specific machinery and machines, pre-planned actions, and standardized movements. Most gym activities are executed in a closed and controlled environment. Consequently, considering the nature of most gym training practices, they could be considered closed-skills activities. Indeed, a closed-skill activity is characterized by well-prepared activities with a stable and predictable environment [3,4].

A recent review highlighted that closed-skill activities have a lower impact on inhibitory control and cognitive flexibility in all ages [5]. By contrast, open-skill (OS) sports practiced until the age of 18 years have a positive effect on working memory, cognitive flexibility [6], and neuromotor function, such as manual dexterity [7]. Indeed, the practice of outdoor activities, especially during development, is fundamental for enhancing motor skills [8]. A study that compared tennis players (OS sport) with swimmers (closed-skill sport) and non-athletes detected that inhibitory control is superior for tennis players [9]. The literature suggests that cognitive control might benefit more from training in OS sports [9].

Poorer cognitive function (executive function, processing speed, and slower psychomotor speed) is associated with falls in older adults [10]. Processing speed, more than working memory or emotional functioning, is correlated with falls and resulting injuries in older adults [11]. Widely adopted to evaluate processing speed in older adults are the reaction time tests (simple and choice) [12,13]. A study on people aged from 20 to 99 years detected that for hands and feet, the reaction time and its variation increased with the age [14]. Reaction time is associated with the risk of falling in older adults [15]. An association exists between functional measurements such as reaction time during the choice stepping reaction time test and falls [13,16]. The reaction time performance of older people is also associated with gait performance [17], making these parameters interesting for the assessment. Between the tests to evaluate this parameter, the simple reaction time test discriminates between single and multiple fallers [18]. A study on Austrian gym users detected gaps in knowledge of reaction time training methods and related practices [19]. As highlighted before, most gym activities are closed-skills oriented and in older age this could lead to an increased risk of falls. Another aspect that could increase the risk of falls is reduction in muscle stiffness and tendons of the legs [20]. This is independent of muscle mass and strength [21]. A low gastrocnemius medial head stiffness is associated with lower postural balance ability [22]. A reduction in Achilles tendon stiffness and plantar flexor strength has a negative impact on gait stability [23]. It is therefore also important to study this parameter to better understand the risk of falls.

Considering the previous aspects, the hypothesis is that gym users could present a worse condition in tasks where central processing impacts the motor task, such as in a reaction or muscle stiffness task. Consequently, the objective of the present study was to investigate whether a difference in a simple reaction time and muscle stiffness task in terms of physical performance and superficial electromyography (sEMG) signal exists between gym users and people who practiced an OS sport.

## 2. Materials and Methods

### 2.1. Participants

Young adults were considered for this study. They were recruited from Sport Sciences students of the University of Catania. The participants had similar backgrounds to limit the confounding factors. Participants were reached through flyers and word of mouth. They were included if they were aged between 18 and 26 years, if they were OS sport practitioners or gym users, if they were physically healthy (no musculoskeletal lesions in the previous 6 months or orthopedic problems), and if they had no cognitive impairments (they could understand the protocol and perform it safely). Participants had to have been practicing the activity for at least one year, two times a week. There were no eligibility criteria related to the sport level or the intensity of the training, resistance training exposure, competitive level, or training specificity. Only recreational practitioners were included. This decision was taken to provide a clear overview of the overall physically active population and not of professional athletes.

Participants were included if written informed consent for participation was signed before data collection. Participants were informed about the study protocol and the possible risks and benefits. They were volunteers and were advised that they could leave the study at any time and any moment without problems. They did not receive compensation. The study was approved by the scientific committee of the “Research Center on Motor Activities” of the University of Catania (CRAM-59-2024, 17 July 2024). It was conducted following the Declaration of Helsinki. Data were managed anonymously.

Participants comprised two groups: the gym user group and OS sport group. The gym user group was composed of people who only trained by themselves in terms of fitness with activities in a gym. The OS sport group was composed of athletes practicing different OS-oriented sports. The decision to create only one group made up of different athletes was taken because a study affirmed that OS or closed skills athletes generally have greater sensory-cognitive skills that are connected to the specific sport domain [24].

A formal a priori sample size calculation was not required due to the type of study [25]. However, to enable a better understanding of the number of participants required for future investigations, the sample size was determined using the software G*Power (3.1). The analysis was performed based on feasibility and in accordance with a previous study that investigated postural control in different athletic populations [26]. An effect size of d = 0.70, a probability error of alpha = 0.05, and a power of 0.8 were adopted. Based on these data, a target of 34 participants per group (total number = 68) was considered adequate to detect large effect sizes and to estimate variability for future confirmatory studies.

### 2.2. Study Design

**Figure 1:**
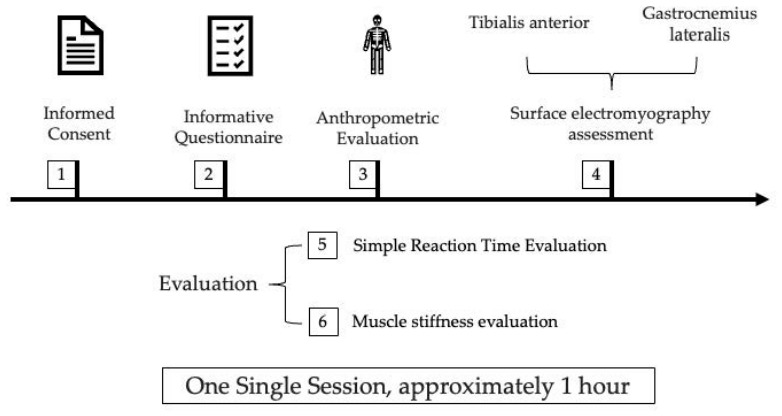
Graphical description of the study design.

After the informed consent signature, an informative questionnaire was proposed to determine participants’ eligibility. Before data collection, participants were evaluated in terms of anthropometric characteristics. Height and bioelectrical impedance analysis data were collected. Before the jump performance, electrodes were set at the tibialis anterior (TA) and gastrocnemius lateralis (GL). A maximum voluntary contraction evaluation for the two muscles tested was proposed in this phase. With the sEMG and electrodes placed, participants performed the simple reaction time task. Finally, participants performed a drop jump test (muscle stiffness); due to the characteristics of the jump, it was impossible to execute sEMG analysis. The graphical description of the study design is presented in Figure 1.

### 2.3. Anthropometric Evaluation

Participants had to not eat breakfast (and not drink coffee or other drinks) at least three hours before the bioelectrical impedance analysis. They also had to avoid main meals in the eight hours before, and heavy exercise or alcohol in the previous twelve hours before the evaluation. The body composition analyzer Tanita (MC-780A; TANITA Corporation, Tokyo, Japan) was used and is valid and reliable [27,28,29,30]. Tanita has 93/42 EEC certification (EU norm for medical devices). The only parameter considered was the body fat percentage (FAT%).

Participants were required to remove all objects containing metal. If this was impossible, participants were excluded from the study. After 10 min in which participants remained seated and completed the questionnaire, they were required to be dressed in shorts and a t-shirt. Once participants were correctly positioned on the instrument, the data were collected.

### 2.4. Surface Electromyography Assessment

**Figure 2:**
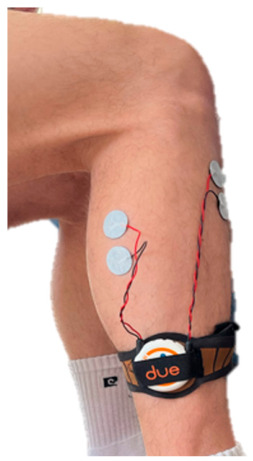
Electrode positioning. The figure shows where the electrodes were positioned for the tibialis anterior and the gastrocnemius lateralis.

The TA and the GL muscles of the dominant leg were evaluated during the reaction time task. The setting is presented in Figure 2. To place the electrodes, the recommendations provided by the group SENIAMs (Development of recommendations for SEMG sensors and sensor placement procedures) were followed [31]. To detect the muscles, previously published guidelines were also followed [32]. For the study, bipolar electrodes of 24 mm with a concentric connector (5pcs) and Ag/AgCl surface (Spes Medica, Battipaglia, Italy) were adopted. A 0.5 cm interelectrode distance was adopted. The first part of the procedure was executed by an investigator with previous experience with sEMG. A two-channel sEMG (Duelite, OT Bioelettronica, Turin, Italy) was adopted. This instrument presents a recording bandwidth of 10–500 Hz with a sampling frequency of 2048 Hz.

Data were analyzed through the OT BioLab software (version 1.8, OT Bioelettronica, Torino, Italy). In the first step, the sEMG signal was root mean square (RMS)-converted and expressed in millivolts (mVs). Data are presented as absolute values and as the percentage of a maximal voluntary contraction. The agonist/antagonist muscle ratio is presented as the relationship between GL and TA.

Before the jumps, immediately after the bioelectrical impedance analysis, participants performed a maximum voluntary contraction test. Participants were seated on a chair that allowed a knee and ankle angle of 90°. The body segments were blocked to make the contraction isometric. An isometric dorsiflexion was adopted to evaluate the maximum voluntary contraction of the TA. An isometric plantar flexion was proposed for the maximum voluntary contraction of the GL. Participants were instructed to reach the maximal contraction gradually, over three seconds, and to keep the maximal contraction for two seconds [33].

### 2.5. Simple Reaction Time Evaluation

The simple reaction time was recorded with the Optojump photocell system (Microgate, Bolzano, Italy). The protocol was proposed by the OPTOJUMP NEXT software (version 1.13.24). It requires participants to get both feet off the ground after a visual stimulation (a green dot appeared on the screen instead of a red one). Participants were advised to react as soon as possible. They stood in front of a screen, within the Optojump bars, feet shoulder-width apart, in a semi-squat position with arms free. The best time of three consecutive stimuli adopted by the participants to react to the visual stimuli recorded by the Optojump photocell system was used for the analysis.

In this test, it was decided not to undergo a familiarization process. As highlighted by the literature [34], immediate repetition could facilitate stimulus identification, consequently impacting the task. However, a researcher showed and demonstrated the test to the participants beforehand to minimize any misunderstanding of the protocol.

Furthermore, due to the lack of the familiarization phase, it was decided to adopt the best time because in this way, if other factors influenced one of the reaction tasks, it would be excluded.

### 2.6. Muscle Stiffness Evaluation

A vertical drop-jump was proposed to measure muscle stiffness. It is an indirect measure of muscle stiffness, but previous studies have adopted it also for this purpose [35,36]. From a box, height 31 cm, participants had to drop down. This height was chosen because previously published studies suggest that 30 cm maximizes the power expression in movement in which there is limited time to apply a force [37,38]. Feet had to be shoulder-width apart, and they could use their arms. Participants had to land within the two bars of the Optojump and jump as high as possible and as quickly as possible [39,40,41]. The ground contact time was recorded for the analysis.

### 2.7. Statistical Analysis

The normality of the data distribution was tested with the Shapiro–Wilk test; α was set at 0.05. Data are expressed as mean and standard deviation. An ANOVA test and a Turkey’s multiple comparison test were adopted to study the differences between the variables FAT%, reaction time, and ground contact time of the gym users and OS-sport groups. Within the group, the Pearson correlation test was adopted between the variables reaction time, years of training, FAT%, and ground contact time.

For the sEMG data of the reaction time task, a Kruskal–Wallis test and the Dunn post hoc test were carried out, considering that the data were not parametric. The parameters investigated for the sEMG were the absolute values of TA and GL, the percentage of the MVC (presented as %TA and %GL), the absolute value of the relationship between GL and TA (presented as GL/TA), and the percentage of the MVC (presented as %GL/TA).

The significance level was set at *p* ≤ 0.05. The statistical analysis was performed through GraphPad Prism 8.0 for Windows (San Diego, CA, USA).

## 3. Results

After the eligibility criteria application, a total of 58 participants were included in the sample. The mean age was 21.9 ± 2.16 years, and the mean weekly training duration was 353.25 ± 135.8 min. The OS sports group comprised 25 athletes. The sports practiced were soccer (nine participants), basketball (four participants), volleyball (five participants), dance (three participants), and handball, running, cycling, and skating (one participant each). The mean age was 21.68 ± 2.06 years, their height was 176.2 ± 8.38 cm, and their weight was 71.96 ± 8.28 kg. The weekly minutes of training were 346.80 ± 150.27. The gym user group contained 33 athletes. The mean age was 22.12 ± 2.26 years, their mean height was 170.36 ± 7.06 cm, and their weight was 67.89 ± 8.28 kg. A mean of 359.70 ± 121.33 min of weekly training was performed by this group. More details are presented in Table 1.

### 3.1. Between-Group Analysis

Following the ANOVA test (*p* < 0.0001) and Tukey’s multiple comparison test, the reaction times of the gym users were found to not show statistically significant differences from the reaction times of the OS sport group (*p* = 0.999; 95% CI: −3.605–+3.623; Mean Diff: 0.009165). The ground contact time of the two groups presented no statistically significant differences (*p* = 0.9999; 95% CI: −3.617–+3.611; Mean Diff: −0.003045). The body composition presented no differences between groups. For FAT%, the gym user group presented a higher fat percentage compared to the OS sport group (*p* = 0.9996; 95% CI: −2.511–+4.718; Mean Diff: 1.103).

### 3.2. Within-Group Analysis

The correlation between the reaction times of the gym users and the OS sport group presents a Pearson coefficient (r) of 0.133 (*p* = 0.518). The reaction times of the gym users present an r of −0.017 (*p* = 0.924) with the years of training, r = 0.440 (*p* = 0.010) with the FAT%, and r = 0.496 (*p* = 0.003) with the ground contact time. For the OS sport group, the reaction times present a Pearson coefficient of −0.225 (*p* = 0.279) with the years of training, r = 0.525 (*p* = 0.007) with the FAT%, and r = 0.489 (*p* = 0.013) with the contact time.

The training experience of gym users presented an r of −0.300 (*p* = 0.090) with FAT% and r = 0.061 (*p* = 0.946) with the ground contact time. The training experience of the OS sport group presented an r of −0.465 (*p* = 0.019) with FAT% and r = −0.157 (*p* = 0.453) with the ground contact time.

The FAT% of gym users presented an r of 0.456 (*p* = 0.008) with the contact time. The FAT% of the OS sport group presented an r of 0.299 (*p* = 0.147) with the ground contact time.

### 3.3. Superficial EMG Analysis

Due to artifacts and the dynamic nature of the task, the analysis of the sEMG was performed on 21 OS sport group members and 27 gym users. All mean and standard deviation data are presented as absolute values and percentages in Table 2. The Kruskal–Wallis test of the sEMG data during the reaction time task, after the Dunn’s multiple comparisons test, presented no statistically significant differences (*p* > 0.05) for the TA values of gym users against the OS sport group (*p* > 0.9999); %TA of gym users against OS sport group (*p* > 0.9999); GL of gym users against OS sport group (*p* > 0.9999); %GL of gym users against OS sport group (*p* > 0.9999); %GL/TA of gym users against OS sport group (*p* > 0.9999); and GL/TA of gym users against OS sport group (*p* > 0.9999).

## 4. Discussion

This study evaluated the differences in the simple reaction time task and muscle stiffness in terms of physical performance and sEMG signal between gym users and people who practiced OS sports. No statistically significant results were detected in the parameters studied. Generally, the OS sport group presented the best results in the reaction time while the gym users performed better in the ground contact time trial, but with small differences and no statistically significantly different results. Unfortunately, the results may have been influenced by different factors; above all, the limited number of participants making up the samples was below the number indicated by the power analysis. It is also important to consider that, despite the small difference in the amount of weekly training between the two groups, 12.81 min, parameters such as training intensity, resistance training exposure, competitive level, and training specifity were not considered. Other limitations are highlighted in the last section of the discussion.

The hypothesis was based on literature which highlighted how the training of the proprioceptive system is fundamental in different sports such as soccer, basketball, taekwondo, fencing, speed skating, running, aerobic gymnastics, swimming, sports dancing, and badminton [42,43]. On the other hand, no studies have been found on gym use and its relationship with neuromuscular functioning. Consequently, the study wanted to better understand neuromuscular functioning in gym users. The results showed a lack of statistically significant differences detected between the two groups in terms of reaction time. In old age, muscles are generally weaker and slower, with a decline in muscle power and an increase in fatigability, especially during high-velocity dynamic tasks [44]. Gym activities can be a feasible solution for the stimulation of the neuromuscular system, as studies on older adults show [45]. From the findings, it is not possible to determine the level of muscles stiffness stimulation in gym users and the OS sports group. However, it can be noted that a high level of power can help in better controlling postural balance [46], preventing falls [47]. Further studies are required to analyze the processing speed and cognitive functions between these two populations in depth. It also serves to highlight how in real life, especially among older adults, executive function becomes central because people usually perform more tasks at the same time [48]. It will be interesting to study the effects of different cognitive loads on motor performance in these two populations.

The analysis demonstrated how simple reaction time and contact time (stiffness) were positively correlated with FAT%. The body FAT%, not the body weight, positively influences the simple reaction time, as is demonstraded by the literature [49]. Body lipid reserves could help in the development of the nervous system [49]. The negative correlation between simple reaction time and years of training is explained by the experience and the higher amount of training of athletes with more experience. The reaction time was positively correlated for both groups with the contact time (stiffness). This could be explained by the neuromuscular system that is involved in both tasks, supporting the efficacy of training in both groups. A recent study detected a positive correlation between agility and reaction time in young soccer players [50], demonstrating how the executive functions are often correlated. Indeed, the ground contact time was slightly positively correlated with the training experience for the gym group, while it was slightly negatively correlated for the OS sport group. The negative correlation could be justified by the experience of the athletes, which can make the difference. The trend in gym users is positive but there is no a statistically significant correlation (r = 0.061). This could indicate that gym users’ reaction times need to be further investigated to better understand and contextualize them.

Study limitations are various. First, the number of people included in the study did not reach the 34 participants suggested for each group by the power analysis. A second important limitation is related to the possible training routine aspects, which were not investigated in depth. Factors such as the training intensity, resistance training exposure, the competitive level and the training specificity were not adequately considered. All these aspects could have an impact on the lack of differences between the groups considered. This study aimed to provide an overview of the topic in general to provide the first feedback to the research community in this area, but future studies should take all these aspects into consideration. The age of the participants is also a confounding factor. The decision to include only young adults was made because the number of gym users has crucially been increasing in recent years, and we thought that this population could be representative. Considering the findings, the study could be replicated with older athletes and those with more training experience. We only tested them during simple reaction time tasks, limiting the study to cognitive function in one process. Furthermore, the methodology adopted in this test could introduce variability and affect reliability, impacting the findings. Future studies could use more specific tests, and also test muscle stiffness assessment. A direct evaluation could increase the quality of the data. It is also important to highlight the inclusion of different heterogeneous sports in one category, not considering the different neuromuscular and cognitive loads. Furthermore, stiffness was evaluated indirectly with the drop jump test; future studies could adopt direct evaluations to obtain more precise values. This study potentially provides some feedback to the scientific community with the hope of starting a research line on gym users.

## 5. Conclusions

No statistically significant differences were detected between gym users and the OS sport group in terms of simple reaction time and stiffness. Also, considering the sEMG, no statistically significant differences were detected. The findings could be influenced by different factors, which makes their interpretation speculative. Further and more structured investigations are required.

## Figures and Tables

**Table 1:** Characteristics of the participants included in the study. Data are presented as mean and standard deviation.

	Sample (Female)	Age (Years)	Weekly Training (min)	m Reaction Time (ms)	Contact Time (ms)	Body Fat (%)
OS sport	25 (5)	21.68 ± 2.06	346.80 ± 150.27	0.5196 ± 0.088	0.2061 ± 0.03	18.32 ± 6.45
Gym users	33 (9)	22.12 ± 2.26	359.70 ± 121.33	0.5104 ± 0.099	0.2092 ± 0.03	17.21 ± 5.09

Note: ms: milliseconds; %: percentage.

**Table 2:** Mean values and standard deviation of the absolute and percentage values of the gastrocnemius lateralis and tibialis anterior of the superficial electromyography evaluation.

	TA		GL		GL/TA	GL/TA
	Absolute Value (mV)	%	Absolute Value (mV)	%		%
Gym user group	19.54 ± 14.22	2.85 ± 1.84	27.59 ± 14.80	7.18 ± 3.73	3.49 ± 2.84	1.72 ± 0.97
Open skills sport group	19.31 ± 16.91	2.94 ± 2.84	28.5 ± 19.03	7.60 ± 4.21	3.22 ± 1.74	1.62 ± 0.6

Note: GL: gastrocnemius lateralis; mV: millivolt; %: percentage; TA: tibialis anterior.

## Data Availability

Mean data and standard deviation are included in the tables in the study.
